# *E. coli* and gut health—For the loser now will be later to win

**DOI:** 10.1111/febs.70326

**Published:** 2025-11-14

**Authors:** Jon O. Lundberg

**Affiliations:** ^1^ Karolinska Institutet Stockholm Sweden

**Keywords:** cancer, nitrate, nitric oxide, nitrite, N‐nitrosamines

## Abstract

In humans, commensal gut bacteria participate in a miniature nitrogen cycle, metabolizing dietary nitrate and nitrite. While these processes can generate harmful N‐nitrosamines, they may also produce beneficial nitric oxide (NO) or ammonium. Hager and colleagues demonstrate that *E. coli*, though representing less than 1% of the gut flora, plays a dominant role in reducing nitrate and nitrite in the gut, potentially detoxifying carcinogenic intermediates by converting nitrite to ammonium. Their findings reveal a beneficial facet of a bacterium otherwise often linked to disease.

## Graphical abstract

In humans, commensal gut bacteria participate in a miniature nitrogen cycle, metabolizing dietary nitrate and nitrite. While these processes can generate harmful N‐nitrosamines, they may also produce beneficial nitric oxide (NO) or ammonium. Hager and colleagues demonstrate that *E. coli*, though representing less than 1% of the gut flora, plays a dominant role in reducing nitrate and nitrite in the gut, potentially detoxifying carcinogenic intermediates by converting nitrite to ammonium. Their findings reveal a beneficial facet of a bacterium otherwise often linked to disease. Image by CDC on Unsplash.

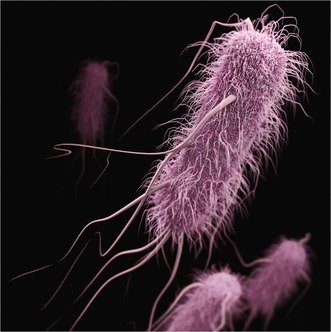

Nitrogen is essential for all life, but the abundant nitrogen gas (N_2_) in the atmosphere is not easily accessible for incorporation into animals and plants due to its extreme unreactivity. In the nitrogen cycle, atmospheric nitrogen is oxidized to higher oxides of nitrogen, thereby providing more reactive nitrogen species that can be used in reactions, ultimately allowing incorporation and the building of amino acids and proteins. In nature, bacteria play a key role by catalyzing several of these reactions. Humans harbor many such bacteria, most of which reside in the gastrointestinal tract. In fact, we have our own mini version of the nitrogen cycle inside [1]. Research performed over the past 60 years or so has shown that commensal bacteria metabolize endogenous and dietary‐derived nitrogen oxide species in a number of ways. Most focus has been on the potential harmful effects of these reactions [2, 3, 4]. Gut commensal bacteria expressing nitrate reductases catalyze the two‐electron reduction in nitrate to nitrite, and further reactions of nitrite can facilitate the formation of N‐nitrosamines, compounds with carcinogenic properties [3, 5]. Therefore, the abundance of nitrate in our everyday diet is causing serious health concerns, and nitrate levels in our food and drinking water are strictly regulated by the authorities. Despite these worries, it has been notoriously difficult to prove a link between dietary nitrate intake and increased incidence of cancer in humans [6]. The effects of nitrate depend heavily on what is generated downstream; if N‐nitrosamines are formed, there is likely reason for concern. If, however, other nitrogen oxides were to be formed, the exact opposite may result!

In 1994, researchers from Sweden and the United Kingdom independently identified the formation of the signaling molecule nitric oxide (NO) from the very same bacterial nitrate‐reductase pathway [7, 8]. Oral bacteria reduce dietary‐derived nitrate to form nitrite, which is further reduced nonenzymatically to NO in the acidic stomach [9]. Moreover, in blood and tissues, systemically absorbed nitrite can also be reduced to NO in processes involving proteins and enzymes. This process, now known as the nitrate–nitrite–nitric oxide pathway, has proven to support a number of host physiological processes, including the regulation of cardiovascular and metabolic function [9, 10, 11, 12].

In this issue of the *Journal*, Hager and colleagues have examined in detail another way by which gut bacteria can help to divert nitrate metabolism away from N‐nitrosamine formation [13]. In this detoxification pathway, the nitrate reductase product nitrite is rapidly shunted into another reaction forming ammonium instead of N‐nitrosamines. The key enzymes studied here are nitrate reductases and nitrite reductases, and the authors have beautifully characterized the various species responsible for these reactions. These enzymes are present in many bacterial species because bacteria utilize nitrate and nitrite for respiration or for incorporation of nitrogen into biomass [1]. As it turns out, *E. coli*, despite constituting only a fraction of the gut bacterial community, is by far the most dominant metabolizer of nitrate and nitrite. Other species, including *Bacteroides* and *Phocaeicola*, also contributed because of their high abundance. Overall, the high activity of these enzymes is expected to result in very low nitrite levels in the lower gut, thereby leaving little room for N‐nitrosamines to be formed locally.

*E. coli* is an interesting bacterium—likely one of the most studied species ever. While it is present in most people, it constitutes less than 1% of our gut bacteria [13]. Most *E. coli* strains are considered harmless and commensal, but there are also pathogenic ones, including enteropathogenic *E. coli* and enterohemorrhagic *E. coli*, that can cause serious gut infections. Even the commensal *E. coli* K‐12 strain can cause disease if it ends up in the wrong place. Indeed, the vast majority of urinary tract infections are caused by this bacterium, and its escape into the bloodstream can cause sepsis. A more recently suggested harmful effect of *E. coli* is more directly related to its use of nitrate for respiration [14]. An inflammatory process in the intestines can result in massive upregulation of an inducible NO synthase in the gut mucosa [15]. This enzyme generates large amounts of NO, and when NO is further oxidized to nitrate, this substrate can be utilized by *E. coli* to outcompete other non–nitrate‐reducing bacteria, possibly leading to overgrowth and disease [14]. It is nice to see that Hager *et al*. now present data to support another, more beneficial side of this bacterium. Certain strains of *E. coli* are classified and even sold as probiotics, an example being *E. coli* Nissle. The mechanisms of any probiotic effects of this bacterium are only partly understood, and it is possible that they involve its clearing of residual nitrite in the large intestine.

In general, one could foresee new therapeutic and preventive strategies involving stimulation of bacterial nitrate and nitrite reduction, either in the oral cavity to enhance systemic NO signaling via the nitrate–nitrite–nitric oxide pathway, or in the lower gut to prevent local N‐nitrosamine formation. This could be done by simple ingestion of nitrate (prebiotics), promoting the strains that utilize nitrate, or by direct delivery of bacteria (probiotics). At the same time, one should also be cautious about disturbing the normal gut flora, especially keeping in mind the above‐described harmful chemistry that can arise from the very same substrates.

In summary, Hager et al. present compelling evidence that a single bacterial species—representing less than 1% of the gut flora and typically associated with disease—can nonetheless play a pivotal role in the detoxification of carcinogens. As Dylan reminds us, ‘*for the loser now will be later to win*’.

## Conflict of interest

JOL is a named inventor on patents relating to the medical uses of inorganic nitrate and nitrite, and a codirector of Heartbeet Ltd.
